# G*α*q Regulates the Development of Rheumatoid Arthritis by Modulating Th1 Differentiation

**DOI:** 10.1155/2017/4639081

**Published:** 2017-01-19

**Authors:** Dashan Wang, Yuan Liu, Yan Li, Yan He, Jiyun Zhang, Guixiu Shi

**Affiliations:** ^1^Molecular Biology Research Center, Key Medical Health Laboratory for Laboratory Medicine of Shandong Province, Department of Laboratory Medicine, Shandong Medical College, Linyi, Shandong 276000, China; ^2^Department of Rheumatology and Clinical Immunology, The First Affiliated Hospital of Xiamen University, Xiamen, Fujian 361003, China; ^3^Key Medical Health Laboratory for Laboratory Medicine of Shandong Province, Department of Laboratory Medicine, Shandong Medical College, Linyi, Shandong 276000, China

## Abstract

The G*α*q-containing G protein, an important member of *G*_q/11_ class, is ubiquitously expressed in mammalian cells. G*α*q has been found to play an important role in immune regulation and development of autoimmune disease such as rheumatoid arthritis (RA). However, how G*α*q participates in the pathogenesis of RA is still not fully understood. In the present study, we aimed to find out whether G*α*q controls RA via regulation of Th1 differentiation. We observed that the expression of G*α*q was negatively correlated with the expression of signature Th1 cytokine (IFN-*γ*) in RA patients, which suggests a negative role of G*α*q in differentiation of Th1 cells. By using G*α*q knockout (*Gnaq−/−*) mice, we demonstrated that loss of G*α*q led to enhanced Th1 cell differentiation. G*α*q negative regulated the differentiation of Th1 cell by modulating the expression of T-bet and the activity of STAT4. Furthermore, we detected the increased ratio of Th1 cells in* Gnaq−/−* bone marrow (BM) chimeras spontaneously developing inflammatory arthritis. In conclusion, results presented in the study demonstrate that loss of G*α*q promotes the differentiation of Th1 cells and contributes to the pathogenesis of RA.

## 1. Introduction

Guanine nucleotide-binding proteins (G proteins) are the most widely used signal transducers in mammalian cells. G proteins transmit signals from ligand activated G protein coupled receptors (GPCRs) to effector proteins and then regulate many biological functions [1]. GPCR ligands include numerous hormones, neurotransmitters, peptides, small proteins, and lipid molecules. Accordingly, biological functions mediated by G proteins and GPCRs are diverse, including behavioral, sensory functions, appetite control, metabolism, development, inflammation, and chemotaxis [2]. The heterotrimeric G proteins are composed of *α*-subunit that binds and hydrolyzes GTP, as well as *β*- and *γ*-subunits that form a functional complex. The *α*-subunits of G proteins are highly specialized and induce many different downstream signals. Based on the sequence similarity of their *α*-subunits, G proteins can be classified into four subfamilies: Gi, Gs, Gq/11, and G12/13. G*α*q, which is the *α*-subunit Gq protein, is encoded by* GNAQ* [3]. The G*α*q containing G protein initially attracted our attention for its important role in cardiovascular system in 1990s [4]. In recent years, studies have also demonstrated that G*α*q are involved in immune regulation and autoimmune disease. Our previous studies reported that G*α*q regulated migration of dendritic cells and survival of B cells and T cells [5–7]. More important,* Gnaq*−/− bone marrow (BM) chimeras with G*α*q deficiency only in their immune system could spontaneously develop symptoms of arthritis similar to RA [6]. We also reported that the protein and mRNA expression levels of G*α*q in peripheral blood lymphocytes (PBLs) of rheumatoid arthritis (RA) patients were significantly lower compared with healthy controls [8]. These results indicated a critical role of G*α*q in the pathogenesis of RA.

Rheumatoid arthritis (RA) is the most common systemic autoimmune disease characterized by chronic inflammation of joint synovial tissue and subsequent destruction of associated bone, cartilage, and soft tissues [3]. Although the etiology of RA is still not fully understood, T cells are thought to play a central role in joint inflammation and disease progression [9, 10]. Among of them, T helper 1 (Th1) cells have been found to play an important role in RA in several studies. Th1 cells infiltrate the synovium, secret proinflammatory cytokines, and promote macrophage and neutrophil infiltration [11–13]. Our previous study indicated that G*α*q is associated with RA. However, how G*α*q is involved in the initiation and development of RA is not fully studied. In the current study, we will study the role of G*α*q in Th1 cell differentiation and RA. First, we studied the relationship between G*α*q and hallmark Th1 cytokine (IFN-*γ*) in RA patients. Then, we investigated the role of G*α*q in Th1 differentiation and inflammatory arthritis by using* Gnaq−/−* mice. We found that G*α*q was negatively associated with signature Th1 cytokine (IFN-*γ*) in RA patients, which suggested that G*α*q might be involved in Th1 cells differentiation. In consistent with the result we observed in RA patients, the percentage of Th1 cells was significantly increased in* Gnaq*−*/*− BM chimeras which spontaneously developed inflammatory arthritis. Moreover, our results showed that the deficiency of G*α*q heightened the differentiation of Th1 cells via T-bet and STAT4 by using* Gnaq−/−* mice. Here we show that G*α*q might be involved in Th1 cells differentiation and further participate in the pathogenesis of RA.

## 2. Material and Methods

### 2.1. Patients

A total of 30 RA patients fulfilling the American College of Rheumatology 1987 revised criteria [14] were recruited from the outpatient clinic of the Department of Rheumatology and Clinical Immunology, The First Affiliated Hospital of Xiamen University. 30 sex-matched and age-matched healthy volunteers were recruited as healthy controls. The study was done after obtaining written informed consent of patients and approval of the Ethics Committee of The First Affiliated Hospital of Xiamen University. The demographic and clinical features of healthy controls and patients with RA were summarized in Table 1.

### 2.2. Animals

C57BL/6J and* Gnaq*−*/*− mice (*N* > 5 backcrossed to C57BL/6J) were maintained in a pathogen-free animal facility of Xiamen University and used between 6 and 8 weeks of age.* Gnaq−/−* mice are difficult to study as they are born runted and exhibit motor defects. In order to analyze the role of G*α*q in development of arthritis, we generated BM chimeric mice. BM chimeras were generated by irradiating recipient mice with a split dose of 800 Rads and then reconstituting the recipients with 1 × 10^6^ BM cells from C57BL/6 or* Gnaq−/−* donors. All experimental procedures involving mice were approved by the Animal Care and Use Committee of Xiamen University.

### 2.3. Blood Samples

Peripheral blood samples from RA patients and healthy volunteers were collected into collection tubes containing 0.2 mL sodium heparin. Peripheral blood mononuclear cells (PBMCs) were isolated from peripheral blood samples by standard density-gradient centrifugation using Ficoll-Paque Plus (Axis-Shied).

### 2.4. T Cell Purification and In Vitro Th1 Cells Induction

Spleens from WT and* Gnaq*−*/*− mice were passed through a fine nylon mesh to obtain single cell suspension. Red blood cells (RBCs) were lysed using ACK lysis buffer. CD4^+^CD62L^+^ cells were isolated using a negative selection step and a positive selection step. First, CD4^+^ T cells were purified using negative selection with biotinylated Abs against B220, MHC-II, CD8, CD49b, CD11c, and CD11b (eBiosciences); second, CD4^+^CD62L^+^ cells were isolated and then by positive selection using biotinylated Abs against CD62L (eBioscience), followed by streptavidin conjugated magnetic beads (Miltenyi) [15]. Purity, as assessed by flow cytometry, was >92%. Purified naïve CD4^+^ T cells were stimulated with precoated anti-CD3/CD28 (3 *μ*g/mL) for 5 days, with mouse IL-12 (20 ng/mL), mouse IL-2 (20 ng/mL) (PeproTech), and anti-IL-4 (10 *μ*g/mL) (eBioscience) added to the cultures [16].

### 2.5. Flow Cytometry and Intracellular Staining

Cells were collected and stimulated with PMA (25 ng/mL) and ionomycin (1 *μ*g/mL) in the presence of 1 *μ*g/mL monesin (Sigma-Aldrich) for 4 hours. Cells were then fixed, permeabilized by using Fixation/Permeabilization Solution Kit (eBioscience) according to the manufacturer's instructions, and stained with FITC-conjugated anti-IFN-*γ*, PE-conjugated anti-phospho-STAT4, or PE-cy7-conjugated anti-T-bet (eBioscience). In order to stain Th1 cells in mice, single cell suspension was deprived from spleen of WT and* Gnaq−/−* chimeric mice, stimulated with PMA, ionomycin, and monensin for 4 hour. After culture, cells were stained with PE-conjugated anti-CD4, followed by intracellular staining with FITC-conjugated anti-IFN-*γ*. Cells were analyzed on Cytomic FC500 (Beckman Coulter), and data were analyzed using FlowJo (Tree Star).

### 2.6. Real-Time PCR Analysis

Total RNA was isolated from PBMCs using TRIzol (Invitrogen). Complementary DNA was synthesized using reverse transcription reagent kits according to manufacturer's instructions (Bio-Rad). The expression levels of IFN-*γ* and G*α*q were determined by real-time quantitative PCR. A 10 *μ*L SsoFast EvaGreen PCR reaction system was used. It includes 2 *μ*L of cDNA, 2.6 *μ*L ddH2O, 0.2 *μ*L of sense primer, 0.2 *μ*L of antisense primer, and 5 *μ*L SsoFast EvaGreen Supermix (Bio-Rad). The PCR reaction conditions were as follows: 95°C for 1 min, then 40 cycles of 95°C for 10 s, 60°C for 10 s, and 72°C for 10 s. Reactions were performed with iQ™5 real-time PCR Detection Systems (Bio-Rad). Target gene expressions were normalized to GAPDH and relative expression was calculated using the 2^−ΔΔCt^ method. The following primers were used:* GNAQ*, 5′-GTTGATGTGGAGAAGGTGTCTG-3′ and 5′-GTAGGCAGGTAGGCAGGGT-3′;* IFNG*, 5′-GATGACTTCGAAAAGCTGACTAATTATTC-3′ and 5′-GTTCAGCCATCACACTTGGATGAG-3′* GAPDH*, 5′-GTGAACCATGAGAAGTATGACAAC-3′ and 5′-CATGAGTCCTTCCACGATACC-3′.

### 2.7. Enzyme-Linked Immunosorbent Assay (ELISA)

The concentration of mouse IFN-*γ* was detected using commercially available ELISA kits according to the manufacturer's instructions (BioLegend). Absorbance was measured with an ELISA microplate reader at 450 nm.

### 2.8. Statistical Analysis

Data were analyzed with Prism 5.01 software (GraphPad Software). Statistical differences between WT and* Gnaq−/−* groups were determined by Student's *t*-test. Statistical differences between healthy volunteers and RA were determined by Mann–Whitney *U* test. The correlation between G*α*q and IFN-*γ* was analyzed using Spearman test. *P* value < 0.05 was considered to be statistically significant. 

## 3. Results

### 3.1. G*α*q and IFN-*γ* Were Negatively Correlated in RA Patients

**Figure 1:**
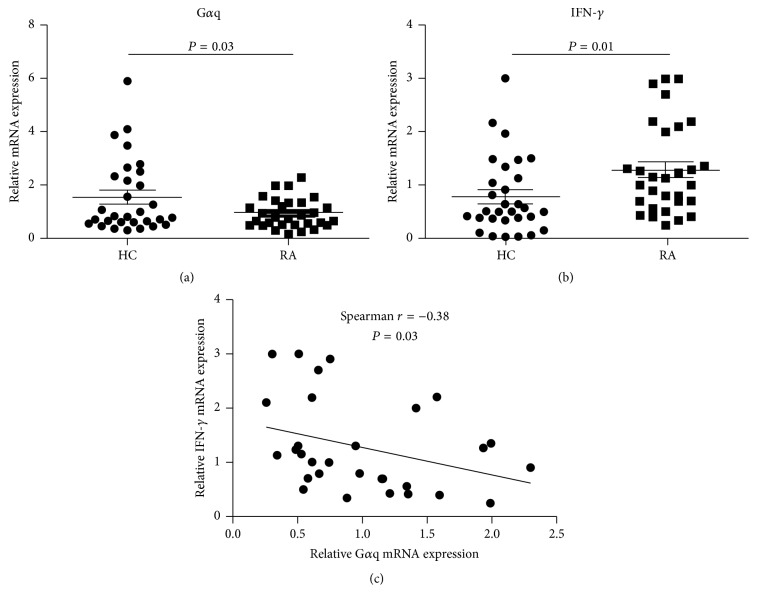
Correlation of mRNA expression level of G*α*q and IFN-*γ*. The mRNA expression of G*α*q and IFN-*γ* was detected by real-time PCR. Relative (a) G*α*q and (b) IFN-*γ* mRNA expression in PBMCs from patients with rheumatoid arthritis (RA; *n* = 30) and healthy controls (HC; *n* = 30). Bars show the mean and standard deviation (SD). *P* value was determined by Mann–Whitney test. (c) The correlation between G*α*q mRNA expression level and IFN-*γ* mRNA expression level in RA patients (*n* = 30) was determined by using Spearman test.

A paper of our group has reported that expression levels of G*α*q were significantly decreased in RA patients and negatively correlated with disease activity [8]. Our previous results also showed that* Gnaq−/−* BM chimeric mice spontaneously developed inflammatory arthritis, indicating that G*α*q might be involved in development of RA [6]. Th1 cell is recognized as a main effector cell in RA progression [17, 18]; thus, whether G*α*q can regulate Th1 cell response and further participates in development of RA attracts our interests. We first investigated the association of G*α*q and hallmark Th1 cytokine (IFN-*γ*) in RA patients. We detected G*α*q and IFN-*γ* mRNA expression in PBMCs from 30 RA patients and 30 healthy controls by real-time PCR. Results showed that G*α*q mRNA expression was significantly decreased and IFN-*γ* mRNA expression was significantly increased in RA patients compared to healthy controls (Figures 1(a) and 1(b)). Moreover, we found a negative correlation between expression level of G*α*q and IFN-*γ* (Figure 1(c)). These data demonstrate that G*α*q was negatively associated with signature Th1 cytokine (IFN-*γ*) in RA patients.

### 3.2. Loss of G*α*q Enhanced the Differentiation of Th1 Cells

**Figure 2:**
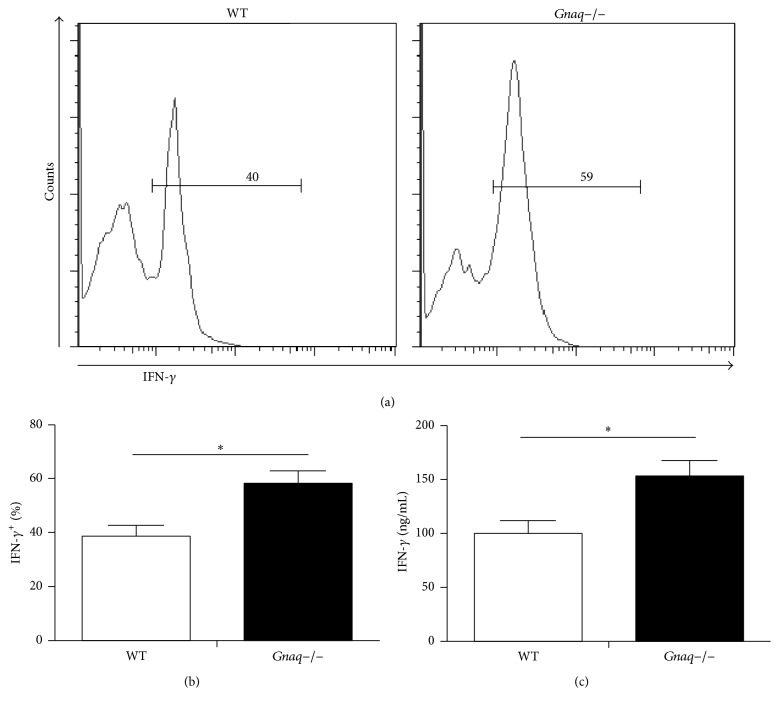
Loss of G*α*q enhances the differentiation of Th1 cells. Purified naïve CD4^+^ T cells from WT and* Gnaq−/−* mice were stimulated with anti-CD3/CD28 (3 *μ*g/mL), in the presence of mouse IL-12 (20 ng/mL), mouse IL-2 (20 ng/mL), and anti-IL-4 (10 *μ*g/mL) for five days. Cells were harvested and analyzed. (a) WT and* Gnaq−/−* CD4^+^ T cells were stimulated with PMA, ionomycin, and monensin, fixed, permeabilized, and stained with FITC-conjugated anti-IFN-*γ*, followed by flow cytometry. (b) The percentage of IFN-*γ*^+^ cells was calculated. (c) IFN-*γ* secretion was detected by ELISA. Cultured CD4^+^ T cells were harvested, adjusted to same concentration, and stimulated by anti-CD3/CD28 (1 *μ*g/mL) for 24 hours. Supernatants were collected for ELISA assay. All data are presented as mean ± SD; ^*∗*^*P* < 0.05, *n* = 3. The result is representative of three independent experiments.

The result presented above encouraged us to study whether G*α*q regulates Th1 cell differentiation. In order to address this question, we used G*α*q knockout (*Gnaq−/−*) mice. Naïve CD4^+^ T cells were purified from spleen of* Gnaq−/−* and WT mice and incubated under Th1 differentiation condition. After 5-day culture, cells were harvested and analyzed by flow cytometry (Figure 2(a)). Intracellular staining showed higher frequency of IFN-*γ*+ cells in* Gnaq−/−* CD4^+^ T cell than WT group (Figure 2(b)). We also measured the level of IFN-*γ* in supernatant by ELISA. Cultured CD4^+^ T cells were harvested, adjusted to same concentration, and stimulated by anti-CD3/CD28 (1 *μ*g/mL) for 24 hours. Supernatants were collected and cytokine concentrations were measured by ELISA assay. Result demonstrated that secretion level of IFN-*γ* was also much higher in* Gnaq−/−* CD4^+^ T cell (Figure 2(c)). These results showed that G*α*q regulates Th1 differentiation.

### 3.3. Absence of G*α*q Heightened the Expression of T-Bet and p-STAT4 in CD4^+^ T Cells

**Figure 3:**
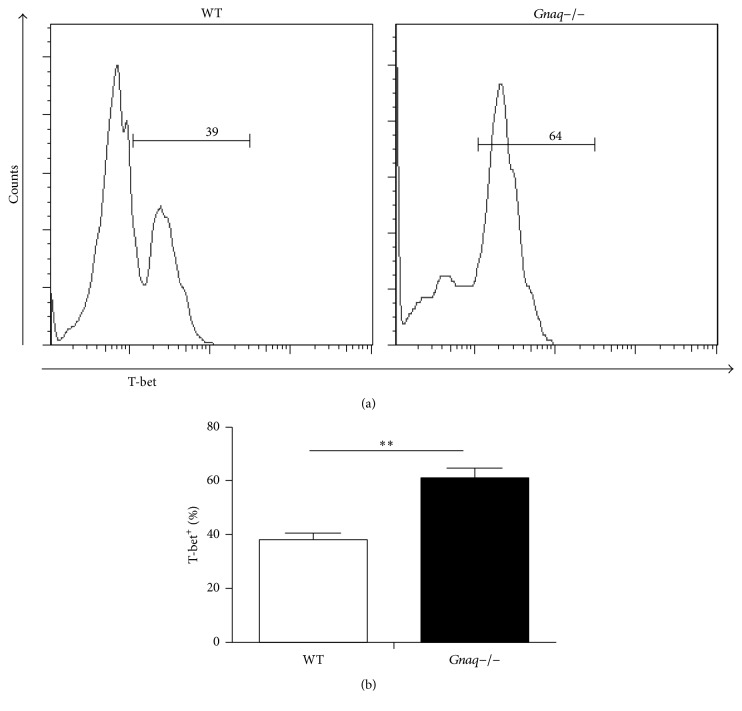
Loss of G*α*q enhances the expression of T-bet. (a) Purified naïve CD4^+^ T cells from WT and* Gnaq−/−* mice were stimulated with anti-CD3/CD28 (3 *μ*g/mL), in the presence of mouse IL-12 (20 ng/mL), mouse IL-2 (20 ng/mL), and anti-IL-4 (10 *μ*g/mL) for five days. Cells were harvested, fixed, permeabilized, and stained with PE-cy7-conjugated anti-T-bet and analyzed by flow cytometry. (b) The percentage of T-bet^+^ cells was calculated. All data are presented as mean ± SD; ^*∗∗*^*P* < 0.05, *n* = 3. The result is representative of three independent experiments.

**Figure 4:**
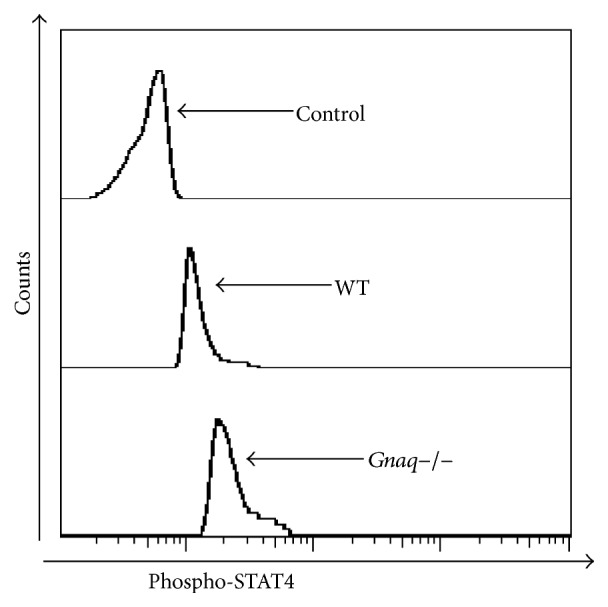
Loss of G*α*q enhances the activation of STAT4. Purified naïve CD4^+^ T cells from WT and* Gnaq−/−* mice were stimulated under Th1 induction condition for 10 minutes. Cells were harvested, fixed, permeabilized, and stained with PE-conjugated anti-p-STAT4 and analyzed by flow cytometry. Data are representative of three independent experiments with similar results.

Results presented above identified a negative role of G*α*q in Th1 differentiation. T-bet, a Th1-specific T box transcription factor that controls the expression of IFN-*γ*, is a critical regulator for Th1 cell differentiation [19]. To explore underlying mechanism of the regulation of G*α*q in Th1 differentiation, we next detected the status of T-bet in WT and* Gnaq−/−* CD4^+^ T cells under Th1 polarizing condition. After 5 days of induction, cells were harvested and expression of T-bet was analyzed by flow cytometry (Figure 3(a)). Result showed that expression level of T-bet was dramatically increased in* Gnaq−/−* CD4^+^ T cells compared with WT CD4^+^ T cells (Figure 3(b)). As STAT4 is also a critical factor in Th1 differentiation, we further measured the phosphorylation of STAT4 by flow cytometry. The level of phospho-STAT4 was obviously higher in* Gnaq−/−* CD4^+^ T cells than WT controls (Figure 4). Therefore, results demonstrate that G*α*q regulates Th1 cell differentiation by modulating T-bet and STAT4 in* Gnaq−/−* mice.

### 3.4. Percentage of Th1 Cells Was Increased in* Gnaq−/−* BM Chimeras Spontaneously Developing Arthritis

**Figure 5:**
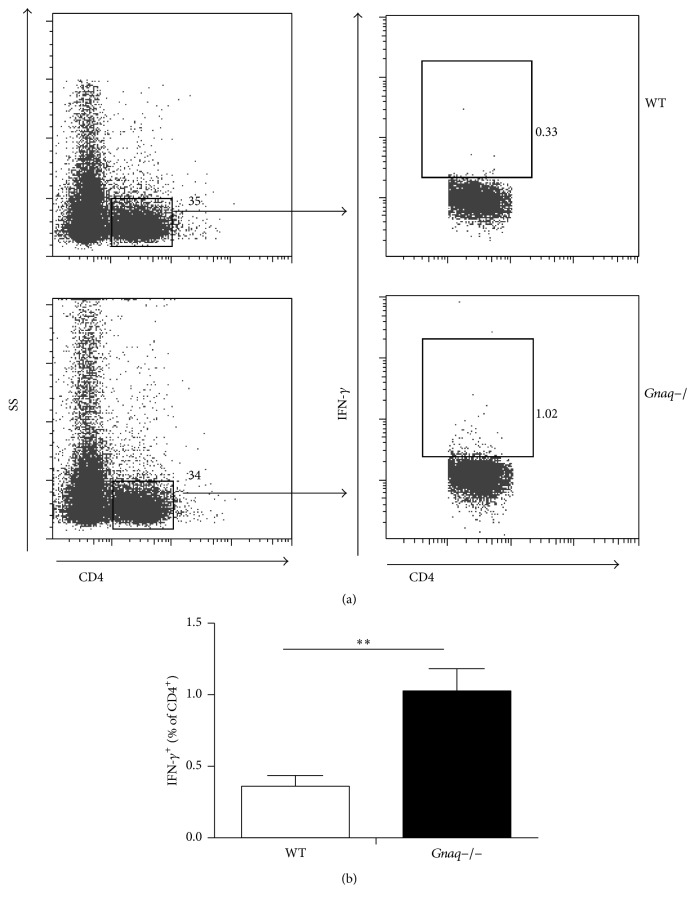
The percentage of Th1 cells is increased in* Gnaq−/−* BM chimeras suffering from inflammatory arthritis. (a) Single cell suspension was deprived from spleen of* Gnaq−/−* BM chimeras suffering from inflammatory arthritis and WT controls, stimulated with PMA, ionomycin, and monensin for 4 hours. After culture, cells were stained with PE-conjugated anti-CD4, followed by intracellular staining with FITC-conjugated anti-IFN-*γ*, and analyzed by flow cytometry. Gated on CD4^+^ cells. (b) The percentage of Th1 cells is presented as mean ± SE, ^*∗∗*^*P* < 0.05, *n* = 3. The result is representative of three independent experiments.

We have identified a negative correlation between G*α*q and hallmark Th1 cytokine (IFN-*γ*) in RA patients and a negative role of G*α*q in Th1 cell differentiation in* Gnaq−/−* mice. Meanwhile, our previous result has demonstrated that* Gnaq−/−* BM chimeric mice can develop symptoms of arthritis similar to RA. Based on these findings, we considered that it is important to determine whether homeostasis of Th1 cell is disturbed in* Gnaq−/−* mice.* Gnaq−/−* mice are difficult when used in the current study as they are born runted and exhibit motor defects. In order to analyze the role of G*α*q in development of arthritis, we generated BM chimeric mice. As previous study,* Gnaq−/−* BM chimeras spontaneously developed symptom of inflammatory arthritis 3.5 months after BM reconstruction. We measured the percentage of Th1 cells in the spleen of autoimmune prone* Gnaq−/−* BM chimeras and WT BM chimeras 3.5 months after BM reconstruction by flow cytometry (Figure 5(a)). Single cell suspension was deprived from spleen of WT and* Gnaq−/−* BM chimeras, stimulated with PMA, monensin, and ionomycin for 4 hours. After culture, cells were stained with PE-conjugated anti-CD4, followed by intracellular staining with FITC-conjugated anti-IFN-*γ*. Results showed that the percentage of Th1 cells was significantly increased in* Gnaq−/−* BM chimeras compared with WT BM chimeras (Figure 5(b)). The spontaneously developed arthritis in* Gnaq−/−* BM chimeras might be partially attributed to increased Th1 response.

## 4. Discussion

In this study, we explored the role of G*α*q in Th1 differentiation and development of RA. We found that expression level of G*α*q and hallmark Th1 cytokine (IFN-*γ*) was negatively related in RA patients and percentage of Th1 cells was significantly increased in* Gnaq−/−* BM chimeras spontaneously developing arthritis. Furthermore, we demonstrated that G*α*q negatively regulated Th1 differentiation by modulating T-bet and STAT4 in* Gnaq−/−* mice.

G*α*q has been identified as an important factor in immune regulation in several studies. Moreover, we have showed that expression level of G*α*q was significantly decreased in RA patients and loss of G*α*q in mice leads to autoimmune arthritis [6, 8]. However, the exact mechanism of how G*α*q is involved in the pathogenesis of RA has not been fully studied. RA is a chronic autoimmune disease characterized by joint synovial inflammation and destruction of the surrounding tissue [20]. Although it is still unclear how RA is initiated, T cells, particularly CD4^+^ T helper (Th), are considered to be critical to initiation and maintenance of this disease by secreting proinflammation cytokines that regulate immune reactivity and synovial inflammation [21, 22]. The two CD4^+^ Th subsets, Th1 cells which secrete IFN-*γ* as their hallmark cytokine and Th17 cells which secret IL-17, have been recognized as critical factors in the pathogenesis of RA [23]. Although the newly discovered Th17 cells have been proved to be critical in the pathogenesis of RA, the important role of Th1 cannot be dismissed. Numerous studies have demonstrated that Th1 cells play an important role in promoting inflammation in RA [24]. In our study, we found a negative correlation between expression level of G*α*q and IFN-*γ*, which suggests that G*α*q might negatively regulate Th1 differentiation in RA patients. A paper of our group reported that* Gnaq*−*/*− BM chimeras would exhibit manifestations of inflammatory arthritis 3.5 months after reconstitution. Besides, autoantibodies such as anti-nuclear Ab (ANA) and anti-double-stranded (ds) DNA Ab were significantly increased in* Gnaq*−*/*− BM chimeras [6]. The symptom of inflammatory arthritis in* Gnaq*−*/*− BM chimeras is similar to RA in humans. What is more,* Gnaq*+*/*− mice have been used as mice model of autoimmune disease in a recent paper [25]. In line with results we observed in RA patients, the percentage of Th1 cells was significantly increased in spleen of* Gnaq*−/− BM chimeras spontaneously developing inflammatory arthritis. Taken together, these observations indicated that G*α*q negatively regulates Th1 differentiation and partially contributed to the pathogenesis of RA.

The induction and maintenance of each CD4^+^ Th cell are mainly determined by cytokine environment at the time of naïve T cell activation [26]. Th1 cell polarization is usually induced in the presence of IL-12 and IFN-*γ*, which activate the expression of the master regulator transcription factors, such as T-bet and STAT4 (signal transducer and activator of transcription 4) [27]. T-bet can remodel the* Ifng* gene and promote IFN-*γ* expression [28]; besides it can upregulate IL-12R*β*2 expression [29], leading to enhanced Th1 cell expansion in response to IL-12. STAT4 is also an essential factor in regulation IL-12 signals and Th1 differentiation; the importance of this molecular in Th1 differentiation is demonstrated by STAT4^−/−^ mice [30]. Both STAT4 and T-bet are needed for maximal IFN-*γ* production and while loss of either one will lead to disturbed IFN-*γ* production [31]. In this study, our results showed that expressions of p-STAT4 and T-bet were both upregulated in* Gnaq*−/− CD4^+^ T cells. G*α*q might regulate Th1 differentiation by modulating T-bet and STAT4. Nuclear factor of activated T cells (NFAT), a Ca^2+^ dependent transcription factor family, has been shown to be important in T cell differentiation. Sustained NFAT signaling promoted CD4^+^ T cells differentiate to Th1 cells in Th1-skewing conditions [32]. Another study demonstrates that STAT4 enhanced Th1 differentiation IFN-*γ* expression by upregulating the binding of activator Protein-1 (AP-1) to the IFN-*γ* promoter sequence [33]. Interestingly, one previous study reported that G*α*q knockdown in T cells significantly increased NFAT and AP-1 activity [34]. These data suggest that G*α*q might also regulate Th1 cell differentiation via NFAT/AP-1 signaling pathway.

## 5. Conclusions

Taken together, we demonstrated that G*α*q inhibited the differentiation of Th1 cells and participated in pathogenesis of RA. G*α*q might regulate Th1 cell differentiation via modulating activity of STAT-4 and T-bet. This study provides a new insight into the pathogenesis of RA and suggests a novel therapeutic target for autoimmune disease.

## Figures and Tables

**Table 1:** Demographic and clinical characteristics of the patients with rheumatoid arthritis (RA) and healthy control subjects.

	RA patients (*n* = 30)	Healthy controls (*n* = 30)
Age, mean (range) years	46.1 (33–75)	45.5 (28–64)
Male : female	5 : 25	8 : 22
C-reactive protein (mg/L)	15.9 (1.1–90.6)	—
Rheumatoid factor (IU/mL)	192.0 (6–943)	—
DAS28^*∗*^ mean (range) score	2.99 (1.12–5.07)	—

^*∗*^DAS28 = 28 joints' disease activity score.
